# Clinical Performance of Semi-Automated Spectral-Domain Optical Coherence Tomography Angiography

**DOI:** 10.3390/jcm13216301

**Published:** 2024-10-22

**Authors:** A. Yasin Alibhai, Mary K. Durbin, Huiyuan Hou, Srinivas R. Sadda, Dennis M. Marcus, Timothy T. You, Nevin W. El-Nimri, Lukas Huebschmann, Nadia K. Waheed

**Affiliations:** 1 Boston Image Reading Center, Boston, MA 02111, USA; yalibhai@bostonimagereadingcenter.com; 2 Topcon Healthcare, Oakland, NJ 07436, USA; mdurbin@topcon.com (M.K.D.); nel-nimri@topcon.com (N.W.E.-N.); lhuebschmann@topcon.com (L.H.); 3 Doheny Eye Institute, University of California, Los Angeles, CA 91105, USA; ssadda@doheny.org; 4 Southeast Retina Center, PC, Augusta, GA 30909, USA; dmarcus@southeastretina.com; 5 Department of Ophthalmology, Medical College of Georgia, Augusta, GA 30912, USA; 6 Orange County Retina Medical Group, Santa Ana, CA 92705, USA; tyou@ocretina.net; 7 Tufts Medical Center, Boston, MA 02111, USA; nadiakwaheed@gmail.com

**Keywords:** optical coherence tomography angiography, semi-automated, vascular pathologies, clinical performance

## Abstract

**Background/Objectives**: To evaluate the clinical performance of two optical coherence tomography angiography (OCTA) devices, including a semi-automated device, with respect to image quality and pathology detection, with fluorescein angiography (FA) and indocyanine green angiography (ICGA) serving as the reference standards. **Methods**: In this prospective cross-sectional study, normal eyes and those with various retinal and choroidal pathologies were enrolled and underwent OCTA scanning using semi-automated 3D OCT-1 Maestro2 and Cirrus™ HD-OCT 5000 devices, as well as FA/ICGA imaging. OCTA scans and FA/ICGA images were independently graded for image quality and the visibility of prespecified anatomic vascular features, along with the presence or absence of pathology on the OCTA scans and the FA/ICGA images (within regions corresponding to the OCTA scan areas). Positive percent agreement (PPA), defined as the proportion of eyes in which the OCTA demonstrated pathology when the corresponding FA/ICGA showed pathology, and negative percent agreement (NPA), defined as the proportion of eyes in which the OCTA showed no pathology when the FA/ICGA also showed no pathology, were calculated. **Results**: In total, 38 normal eyes and 86 pathologic eyes were enrolled in the study. The majority of images for both devices were considered clinically useful. The PPA and NPA were high for both devices, indicating a good ability to identify disease when present and to rule it out when not present. **Conclusions**: The findings of this study suggest that the semi-automated Maestro2 and Cirrus have comparably good clinical performance, particularly with regard to accuracy when identifying vascular pathologies.

## 1. Introduction

The reference method for assessing the pathologic retinal and choroidal vasculature in daily clinical practice has been, and continues to be, fluorescein angiography (FA) and indocyanine green angiography (ICGA) [1,2,3]. FA and ICGA provide a wealth of clinically relevant information, such as the visualization of retinal and choroidal neovascularization, vascular leakage and staining, and capillary non-perfusion [3,4]. The field of view of this technique is limited only by the capabilities of the fundus camera in use, allowing for the examination of areas peripheral to the macula depending on the technical properties of the device [4]. However, the two-dimensional nature of dye-based angiography limits its ability to investigate and localize pathological features within the three-dimensional structure of the retina, a capability made possible by optical coherence tomography (OCT). In addition, the quality of the FA/ICGA images can be significantly influenced by the effectiveness of the imaging process, as capturing high-quality images within the brief time window can be challenging. In contrast, OCT can offer an advantage in this regard by providing high-quality images without the same time-sensitive constraints.

Further developments in OCT technology have resulted in optical coherence tomography angiography (OCTA), a fast and non-invasive tool that allows for imaging the microvasculature of the retina and the choroid with a depth resolution in tissue [5]. Motion contrast or blood flow can be identified using temporal decorrelation or changes in reflectivity in OCT B-scans repeated at the same location [5]. In recent years, understanding of the heterogeneity of retinal capillary perfusion and its clinical implications has improved due to research using this novel technology [6]. OCTA offers several clinical advantages over FA, as it does not require the time-consuming and invasive administration of dye [7]. The clinical applications of OCTA in diabetic retinopathy (DR), such as the detection of intraretinal microvascular abnormalities (IRMAs) and neovascularization (NV), as well as the detection of foveal avascular zone (FAZ) abnormalities and areas of retinal ischemia/capillary dropout (RI/CD), have been described [4,5]. A Delphi consensus by international experts recently recommended the use of OCTA in DR severity reporting and other vascular diseases [8]. The application of OCTA in age-related macular degeneration (AMD), particularly for the diagnosis and monitoring of choroidal neovascularization (CNV), as well as other retinal diseases, has also been extensively described [4,9].

Systematic differences between various OCT devices and their limitations are expected due to the use of different technical setups for image generation and processing [10]. Therefore, understanding how different OCTA devices perform relative to each other is crucial for the precise description and characterization of diseases. This understanding is also important for generalizing databases of annotated OCTA images to include multiple devices found in clinical practice [11]. Furthermore, enabling clinics without skilled operators to capture reliable OCTA data will catalyze the utility of OCTA across eyecare, thereby enabling more clinicians to effectively leverage the clinical benefits of the technology.

This study evaluates the OCTA clinical performance of a semi-automated device, the Maestro2, in terms of image quality, visibility of critical anatomical vascular features, and identification of key pathological vascular features. The performance of the Maestro2 OCTA is compared with that of the Cirrus™ HD-OCT 5000 (Carl Zeiss Meditec, Inc., Dublin, CA, USA) to assess these parameters, with FA/ICGA serving as the reference standard. The Cirrus 5000 and Maestro2 are both spectral domain OCT devices. The Cirrus 5000 scans at 68 k A-scans per second, compared to the Maestro2’s 50 k A-scans per second. The Cirrus 5000 offers 2.0 mm axial depth, while the Maestro2 provides 2.3 mm. Both devices use an 840 nm wavelength and have similar axial resolution. For OCTA analysis, the Cirrus 5000 employs the OMAG algorithm, whereas the Maestro2 uses OCTARA. They differ slightly in slab definitions and pixel spacing. Notably, the Cirrus 5000 automates individual steps, such as polarization and Z-scan positioning, while the Maestro2 automates the entire process after pupil identification [5,12].

## 2. Materials and Methods

This was a multi-center, prospective study conducted at two clinical sites: one in California and the other in Georgia, United States. The study protocol was approved by the Advarra Institutional Review Board (6100 Merriweather Dr, Suite 600, Columbia, MD 21044, USA), and the methodology adhered to the tenets of the Declaration of Helsinki for research involving human subjects, as well as to the Health Insurance Portability and Accountability Act (HIPAA). Enrollment began on 8 January 2021, and ended on 9 November 2022. Written informed consent was obtained from all subjects.

### 2.1. Participants

Subjects enrolled in the study underwent an ocular examination to determine the eligibility of the study eyes. The clinical assessment included best-corrected visual acuity (BCVA), refraction, intraocular pressure (IOP), slit lamp biomicroscopy, and fundus examination.

The overall inclusion criteria for subjects in this study were as follows: 22 years of age or older on the date of informed consent, having the ability to understand the written informed consent, and willingness to participate as evidenced by signing the informed consent.

Normal subjects (Normal Population) had a BCVA of 20/40 or better and normal ocular health in the study eye. Subjects were excluded from the Normal Population if they were noted to have clinically significant findings in the study eye during the clinical examination. If only one eye of a subject met the criteria for the Normal Population, that eye was designated as the study eye. If both eyes met the criteria, the study eye was selected randomly.

Subjects in the retinal disease cohort (Pathology Population) were eligible for inclusion if they had been diagnosed with one or more specified pathologies in the study eye and had a BCVA of 20/400 or better in that eye. The study eyes of the Pathology Population were divided into two subgroups: (a) Vascular pathologies primarily visualized in the superficial and deep en face OCTA slabs, including but not limited to DR, branch retinal vein occlusion (BRVO), central retinal vein occlusion (CRVO), central retinal arterial occlusion (CRAO), macular telangiectasia (MacTel), and sickle cell retinopathy (SCR); and (b) Vascular pathologies primarily visualized in outer retina and choriocapillaris en face OCTA slabs, including but not limited to neovascular AMD (“wet” AMD) and polypoidal choroidal vasculopathy (PCV). The eye with the specific pathology was designated as the study eye. If both eyes were eligible, one eye was randomly selected as the study eye. For subjects who met Pathology Population criteria in one eye and Normal Population criteria in the other eye, both eyes may have been enrolled in the study. For subjects that met the Pathology Population criteria, coexisting pathologies were acceptable in the study eyes.

**Figure 1:**
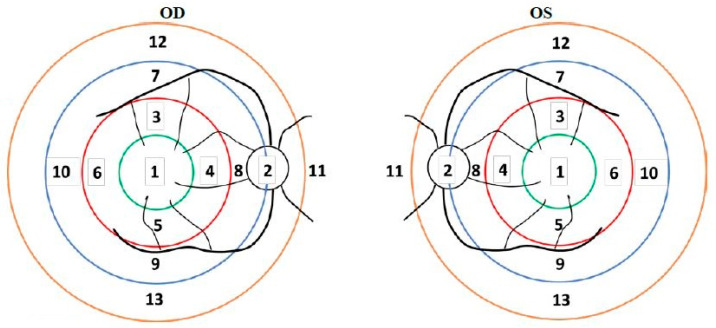
Sketch Map of Retina Regions. The sketch maps delineate 13 retinal regions for both the right and left eyes, with region 1 representing the central macula and region 2 indicating the optic disc. These maps served as a reference for identifying the region of interest (ROI) in the study eye.

During the fundus examination, the investigator identified the primary pathology of interest (PPOI) and the corresponding region of interest (ROI) per scan type in the study eye. ROIs were located and documented based on 13 predefined regions of the retina (Figure 1). Both the PPOI and ROI, as well as any coexisting pathologies, were recorded.

### 2.2. Optical Coherence Tomography Angiography Scans and Other Imaging

Two OCTA devices were used: the Maestro2 (Topcon Corporation, Tokyo, Japan); with Maestro2 software version 2.20 and IMAGEnet6 software version 2.60) and the Cirrus HD-OCT 5000 (Carl Zeiss Meditec, Inc., Dublin, CA, USA; with OCTA software version 11.5.1). Additionally, a TRC- 50DX (Topcon Corporation, Tokyo, Japan) was used for color fundus photography (CFP) and FA/ICGA at each site.

For each OCTA device, three scan types were captured: a 3 mm × 3 mm macular scan, a 6 mm × 6 mm macular scan, and a 4.5 mm × 4.5 mm disc scan. The order of the two devices and three scan types were randomly selected for each study eye. For eyes in the Normal Population, OCTA scans were acquired at the default locations: macular scans centered on the fovea and disc scans centered on the optic disc. For eyes in the Pathology Population, scans were acquired based on the investigator-determined PPOI and corresponding ROI. The operator ensured that the scan field location between the Maestro2 and the corresponding Cirrus scan were matched, particularly when the ROI differed from the default scan locations. A unique Imaging ID (random number) was assigned to each scan type per OCTA device per eye. Clinical site staff reviewed the OCTA scans and selected the first acceptable scan per scan type from each OCTA device based on each device’s respective user manual. If acceptable images could not be acquired through an undilated pupil, dilation was performed. The operator was allowed up to three attempts to acquire an acceptable scan of each scan type after dilation. For the Maestro2 device, the operator selects the pupil and hits capture to initiate the automated setup, but if the operator observed issues in the automated setup, they were able to intervene and use the manual mode, as described in the user manual. Manual segmentation error correction, if needed, was performed by the clinical site staff and documented.

CFP and FA/ICGA images were acquired after the completion of the OCTA scans. A single field encompassing both the optic disc and macula, within a 50° field of view, was captured for all subjects. Pupil dilation was performed when clinically indicated for CFP, FA, and when applicable, ICGA.

Native device software was used to define en face OCTA slabs at various levels of the retinal microvasculature. For the Maestro2, the default slabs included: superficial (from the internal limiting membrane [ILM] to the inner plexiform layer/inner nuclear layer [IPL/INL] + 15.6 µm), deep (from the IPL/INL + 15.6 µm to the IPL/INL + 70.2 µm), outer retina (from IPL/INL + 70.2 µm to Bruch’s membrane [BM]), and choriocapillaris (from BM to BM + 20.8 µm). For the Cirrus, the default slabs included: superficial (from the ILM to the IPL), deep (from the IPL to the outer plexiform layer [OPL]), avascular (from the OPL to the retinal pigment epithelium [RPEfit −70 µm]), and choriocapillaris (from RPEfit + 29 µm to RPEfit + 49 µm). RPEfit refers to the automated segmentation of the retinal pigment epithelium (RPE) in OCT imaging. This fitting process identifies the RPE boundary, which serves as a reference for defining other retinal layers.

### 2.3. Image Grading

An independent reading center, the Boston Image Reading Center (BIRC), conducted image quality grading and identification of anatomic vascular features and pathologies. All graders were masked to the subjects’ demographic information, eye exam details, group assignment, and each other’s grading results throughout the entire study.

Three graders evaluated the Maestro2 and Cirrus OCTA images. The first acceptable scan per scan type and OCTA device, as selected by the clinical site staff, was used for image grading. All OCTA images were graded in a randomized order with an appropriate washout period. OCTA grading, based on corresponding guidelines (Appendix A—Image Grading Criteria), included:

1. OCTA Image Quality: Image quality was graded using a three-level score: Poor, Average, and Good. Both “Good” and “Average” grades were considered clinically useful.
2. Visibility of Key Anatomical Vascular Features: The visibility of the foveal avascular zone (FAZ) border, large-sized vessels, medium-sized vessels, and small-sized vessels/capillaries was graded as Not Visible, Partially Visible, or Visible. Large vessels were not graded in the 3 mm × 3 mm macular scan, and the FAZ was not graded in the disc scan. “Visible” and “Partially Visible” were deemed clinically useful.
3. Identification of Key Pathologic Vascular Features: The identification of microaneurysms (MAs), RI/CD, and CNV was graded as Cannot Grade, No, or Yes. Other pathological vascular features, such as retinal neovascularization (RNV) and macular telangiectasias (MacTel), were noted in the grading form if observed.

**Figure 2:**
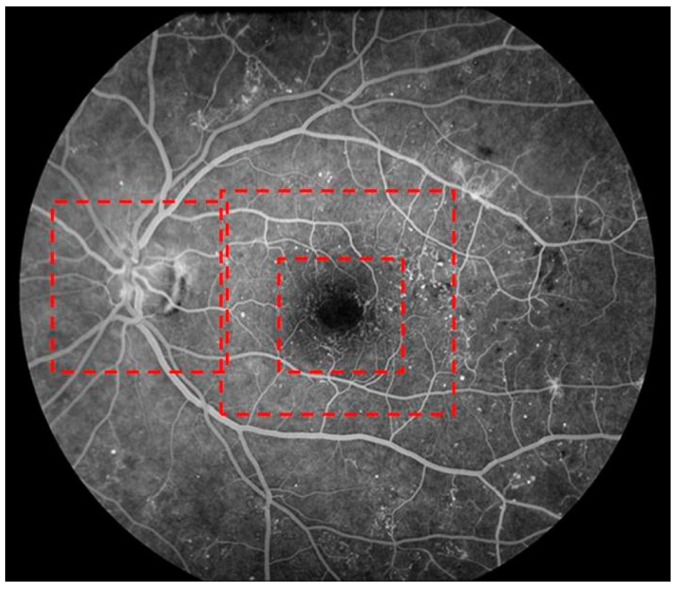
Fluorescein angiography image with demarcation boxes (red boxes) indicating optical coherence tomography angiography scan areas.

FA/ICGA were considered the gold standard for identifying key pathological vascular features. FA/ICGA grading was performed by three graders who were different from those who conducted the OCTA grading. MAs, and CNV within the OCTA scan areas (Figure 2) were graded using the same scale: Cannot Grade, No, or Yes. Other pathological vascular features, such as RNV and MacTel, were noted in the grading form if observed.

The three red demarcation boxes indicate the OCTA scan fields for the three scan types: the left box corresponds to the 4.5 mm × 4.5 mm disc scan; the right outer box corresponds to the 6 mm × 6 mm macular scan; and the right inner box corresponds to the 3 mm × 3 mm macular scan.

CFP images were provided to the graders to accompany both the OCTA and FA/ICGA image grading. The final scores for each grading were determined based on a two-out-of-three agreement. In cases where all three graders disagreed, they convened to discuss and resolve the discrepancies, ultimately providing the final scores.

### 2.4. Statistical Analysis

Descriptive statistics (n, mean, standard deviation [SD]) were used to summarize continuous variables. Frequencies and percentages were used to summarize categorical variables. The proportions of clinically useful images, in terms of OCTA image quality and visibility of key anatomical vascular features, were assessed for Maestro2 and Cirrus devices across different groups and scan types.

Positive percent agreement (PPA) and negative percent agreement (NPA) were calculated for each key pathological vascular feature. PPA represents the ratio of pathology presence identified by consensus OCTA to pathology presence identified by consensus FA/ICGA. NPA represents the ratio of pathology absence identified by consensus OCTA to pathology absence identified by consensus FA/ICGA. All study eyes in the analysis population with pathology identified by consensus FA/ICGA were included in the calculation of PPA and NPA. Eyes with a key pathological vascular feature of “Cannot Grade” from FA/ICGA were excluded from the analysis.

The “response rate” was defined as the percentage of eyes with Maestro2 OCTA grading and corresponding Cirrus OCTA grading in the shaded cells of Table 1 (i.e., (n11 + n12 + n13 + n22)/(n11 + n12 + n13 + n21 + n22 + n23 + n31 + n32 + n33), representing the total number of eyes in the shaded cells divided by the total number of eyes). The response rate was calculated based on matched outcomes, defined as where pathology identification (Yes, No, Cannot Grade) was consistent between OCTA and FA/ICGA. The observed response rate and the exact 95% confidence interval (CI) of the response rate using the binomial distribution were calculated for each scan type and each pathological vascular feature comparison. The analysis was conducted for the Normal Population, the Pathology Population, and for all study eyes combined. Inter-grader agreement rates were evaluated for all grading.

Statistical analyses were performed using SAS software version 9.3 or later (SAS Institute, Cary, NC, USA).

## 3. Results

### 3.1. Demographics and Ocular Characteristics

Among the 41 normal subjects and 93 subjects with retinal pathologies enrolled in the study, 38 eyes from the Normal Population and 86 eyes from the Pathology Population completed the study procedures and were included in the analysis. Enrollment distribution across clinical sites was approximately equal for all populations, with Site 1 enrolling 53.7% of the Normal Population and 50.5% of the Pathology Population. Demographic and ocular characteristics of the study subjects are summarized in Table 2. The mean (SD) age of the overall study population was 54.1 ± 18.4 years, with subjects in the Pathology Population being older than those in the Normal Population. All subjects in the Normal Population were under 65 years of age. The study population was almost evenly distributed by gender, with 45.9% male and 54.1% female. Racial distribution included 17.2% African American and 71.3% white subjects.

Of the 86 eyes with pathology, 51 eyes (59.3%) had retinal conditions that were primarily visualized in the superficial and deep en face OCTA slabs (subgroup 1), and 38 eyes (44.2%) had retinal conditions primarily visualized in the outer retina and choriocapillaris en face OCTA slabs (subgroup 2). In subgroup 1, the most prevalent retinal condition was non-proliferative diabetic retinopathy (NPDR) (22.1%), followed by PDR (10.5%), BRVO (10.5%), CRVO (5.8%), MacTel (4.7%), and SCR (2.3%). In subgroup 2, the most prevalent condition was neovascular AMD (25.6%), followed by PCV (9.3%), myopic CNV (8.1%), central serous chorioretinopathy with neovascularization (2.3%), and traumatic CNV (2.3%). The most common PPOIs were CNV (24.4%), followed by microaneurysms (17.4%), PCV (9.3%), RI/CD (7.0%) and RNV (7.0%).

### 3.2. Clinical Usefulness—Image Quality and Visibility of Anatomical Vascular Features

**Figure 3:**
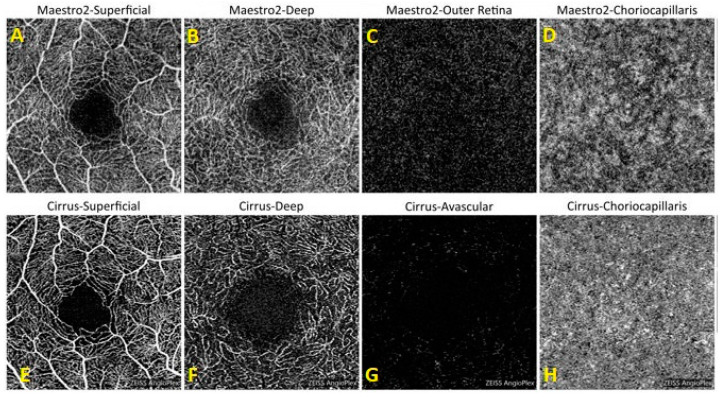
Exemplary default OCTA enface images for 3 mm × 3 mm macular scans from a normal eye for Maestro2 (top row) and Cirrus (bottom row) derived by segmentation of different retina layers and provided to expert OCTA graders. For Maestro2, default slabs include (**A**) Superficial (ILM to IPL/INL + 15.6 µm), (**B**) Deep (IPL/INL + 15.6 µm to IPL/INL + 70.2 µm), (**C**) Outer Retina (IPL/INL + 70.2 µm to BM), and (**D**) Choriocapillaris (BM to BM + 20.8 µm). For Cirrus, default slabs include (**E**) Superficial (ILM to IPL), (**F**) Deep (IPL to OPL), (**G**) Avascular (OPL to RPEfit-70 µm), and Choriocapillaris (RPEfit + 29 µm to RPEfit + 49 µm), (**H**) Choriocapillaris (RPE + 29 µm to RPE + 49 µm). The segmentation is the same for both macula scan sizes (3 mm × 3 mm shown here). The FAZ in the deeper layer is larger for Cirrus than for Maestro. This is likely due to differences in the definition of the deep layer between the two devices.

The majority of images obtained from both normal and pathologic eyes using both devices were considered clinically useful (Table 3). For all eligible study eyes, Maestro2 images were deemed clinically useful in 86.3%, 84.7%, and 82.3% of cases for the three scan types, respectively. In comparison, Cirrus images were clinically useful in 75.8%, 76.6%, and 79.8% of cases, respectively. Inter-grader agreement in image quality grading is summarized in Table S1, showing no variation in image grading among the three graders for the three scan types. Figure 3 shows representative OCTA images obtained from a normal eye using both Maestro2 and Cirrus.

Overall, most images from both devices across the groups and scan types were clinically useful for visualizing anatomic vascular features, ranging from 62.8% to 97.5% across devices and scans (Table 4). Inter-grader agreement in the scores is summarized in Table S2, demonstrating each OCTA image received the same score for each vascular feature from at least two graders.

### 3.3. Identification of Vascular Pathologies

**Figure 4:**
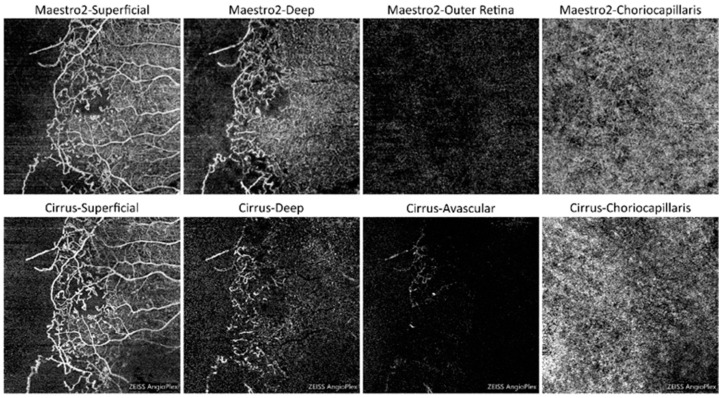
Example case of 6 mm × 6 mm macular OCTA scans from a patient with diabetic retinopathy with OCTA images from both Maestro (**first row**) and Cirrus (**second row**). Features seen include microaneurysms and retinal ischemia/capillary dropout.

**Figure 5:**
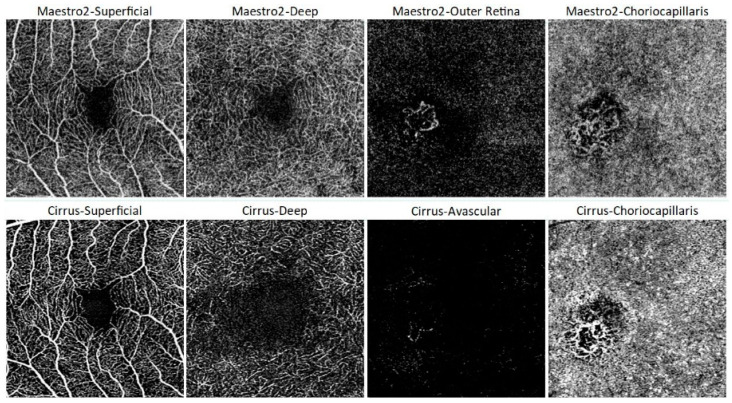
Example of 3 mm × 3 mm scans from a patient with CNV, with en face OCT slabs from Maestro2 in first row, and Cirrus in second row.

Table 5 summarizes the PPA and NPA using the consensus FA/ICGA as the reference, categorized by key pathology, subject population, OCTA scan mode, and OCTA device. Table S3 provides the corresponding 95% CIs for the PPA and NPA values across the different categories. Table 6 summarizes the overall response rates. For all eyes, the PPA for MAs in macular scans was 90.3% for both 3 mm × 3 mm and 6 mm × 6 mm scans with Maestro2, and 92.6% and 88.5% with Cirrus, respectively. The response rate for identifying MAs in the macular scans was 84.4% for the 3 mm × 3 mm scan and 85.2% for the 6 mm × 6 mm scans. The PPA for MAs in the disc scan was lower, at 70.8% for Maestro2 and 66.7% for Cirrus, compared to the macular scans. The response rate for identifying MAs in the disc scan was 80.6%. For all three scan types, the NPA ranged from 95.5% to 100% for both devices. The PPA for RI/CD ranged from 93.1% to 100%, and the NPA ranged from 87.7% to 94.1% for macular scans on both OCTA devices. The response rates for identifying RI/CD in the macular scans were 82.0% for the 3 mm × 3 mm scan and 82.8% for the 6 mm × 6 mm scan. For the disc scan, the PPA for RI/CD was 75.0% for Maestro2 and 81.0% for Cirrus, while the NPA was 92.2% for Maestro2 and 84.4% for Cirrus. The response rate for identifying RI/CD in the disc scan was 78.0%. The PPA for CNV in macular scans (3 mm × 3 mm and 6 mm × 6 mm) was 89.5% and 85.0% for Maestro2, and 87.5% and 83.3% for Cirrus. The corresponding NPAs were 89.7% and 90.6% for Maestro2, and 92.1% and 92.0% for Cirrus. The response rates for identifying CNV in the macular scans were both 84.4%. For the disc scan, the PPA for CNV was 66.7% for Maestro2 and 100% for Cirrus, with NPAs of 99.0% for Maestro2 and 100% for Cirrus. The response rate for CNV identification in the disc scan was 80.5%. The PPA for PPOI in macular scans (3 mm × 3 mm and 6 mm × 6 mm) was 94.1% and 88.6% for Maestro2, and 96.3% and 83.9% for Cirrus. The corresponding NPAs were 83.3% and 82.9% for Maestro2, and 84.6% and 82.3% for Cirrus. For the disc scan, the PPA for PPOI was 73.7% for Maestro2 and 55.6% for Cirrus, with NPAs of 97.6% for Maestro2 and 95.0% for Cirrus. The percentage of total grading agreement is summarized in Table S4. Each OCTA image received the same response for certain pathological vascular features from at least two graders. Figure 4 and Figure 5 show representative OCTA images from Maestro2 and Cirrus for a case with DR and a case with CNV, respectively.

## 4. Discussion

The objectives of this study were to evaluate the clinical performance of the semi-automated Maestro2 OCTA and the Cirrus HD-OCT 5000 OCTA in terms of image quality, visualization of anatomic vascular features, and, most importantly, the identification of key pathologic vascular features, with FA/ICGA as the reference standard. The results indicate that the Maestro2 performs comparably to the Cirrus in generating OCTA images suitable for visualizing retinal and choroidal vasculature and detecting clinically relevant retinal and choroidal vascular pathologies.

Effective clinical interpretation of OCTA technology requires acceptable image quality and an understanding of artifacts. This study found that the semi-automated OCTA data captured with the Maestro2 were comparable to the Cirrus, as evidenced by the large proportion of OCTA images deemed clinically useful and the ability to visualize key anatomic features relevant to clinical care. Both the Maestro2 and Cirrus detected key anatomic and pathologic features, including MAs, RI/CD and CNVs, as well as other vascular pathologies.

MAs are an early and common feature of DR and are important for staging the disease [13]. They can be detected using CFP, FA, and OCTA [1,2,14]. In OCTA, MAs typically appear as outpouchings or bulges of individual vessels [15]. Although it has been noted that MAs imaged with FA may not always be apparent on OCTA or may present differently [10], the results of this study suggest that both devices perform comparably well in identifying MAs in or around the macula. Differences noted in other studies could be due to stagnant erythrocytes blocking the flow or the flow in MAs being below the detection threshold of OCTA [16]. As expected, the PPA for MAs was lower for the disc scan type, with 70.8% for the Maestro2 and 66.7% for the Cirrus compared to the macular scans. MAs predominantly originate from the deep capillary plexus and are generally more severe (in size and number) in this area [17]. Consequently, in OCTA en face images, the identification of MAs in the circumpapillary area can be obscured by projection artifacts from the presence of larger vessels, and a significant portion of the disc scan frame is covered by the optic disc [5]. This effect reduces the utility of OCTA for visualizing MAs in disc scans, though the high response rate of 91% suggests this limitation affects both devices similarly. For all three scan types, the NPA ranged from 95.5% to 100% for both devices. Overall, the findings in this study suggest that Maestro2 and Cirrus have similar accuracy for identifying and ruling out MAs.

OCTA can also be used for the identification and assessment of RI and CD [16]. RI and subsequent CD are key features of various eye diseases, including but not limited to DR, retinal vascular occlusion, and glaucoma. The results of this study suggest that both devices perform similarly well in identifying RI/CD in or around the macula. As with MAs, PPAs are lower for disc scans, though the response rate remains high, suggesting that both devices offer comparable accuracy in detecting RI/CD, with macular scan types being preferred for identifying RI/CD relevant to disease assessment.

CNV is a common pathological condition in several ophthalmic diseases [18]. For all eyes, the PPAs for CNV were over 83% for all scan types across both devices, with corresponding NPAs exceeding 90%, indicating high utility of OCTA for detecting CNV. Notably, one eye was assessed as normal by the site and enrolled in the Normal group, but CNV was identified using OCTA macular scans (both 3 mm × 3 mm and 6 mm × 6 mm) from both devices and was confirmed by FA/ICGA. The subject reported no ocular history. This case highlights the comparable ability of both devices to detect pathology that was not identified during a regular eye examination. The PPA, NPA, and response rate results for CNV identification suggest that the accuracy of the Maestro2 is comparable to that of the Cirrus, with both devices performing similarly in identifying CNV in or around the macula, which is the predominant location for CNV [18].

This study also found that both devices have good and comparable abilities to detect vascular abnormalities associated with pathologies such as MacTel, myopic CNV, retinal neovascularization, and vein occlusions. Although these conditions are less common than DR and AMD, they are characterized by vascular abnormalities that can be imaged with OCTA. Furthermore, the results of this study suggest that both devices perform similarly in identifying pathologies beyond DR and CNV in or around the macula.

The clinical benefit of this study lies in demonstrating that OCTA data can be captured effectively using a device that enables semi-automated data capture, enabling the detection of pathological vascular features, with similar and comparable performance results to an OCTA device that requires manual input from an operator. This is important as it allows clinics without skilled operators to capture reliable OCTA data and clinicians to transfer knowledge from previous experiences to new systems and ensures consistent clinical practices across different devices. Understanding how devices compare is essential for accurate disease diagnosis, treatment monitoring, and achieving a unified understanding of disease among clinicians, particularly when multiple devices are in use.

As we advance towards an era of artificial intelligence (AI) and clinical decision support systems (CDSS), knowing the performance comparability between devices will be vital for ensuring the generalizability and accurate application of algorithms for clinical decision support [19,20]. Large, well-structured and annotated datasets that differentiate between healthy and diseased states are necessary for developing these algorithms [11]. The lack of standardization in OCTA has been noted as a challenge for defining clinical trial endpoints [21]. However, studies like this may help address this issue.

Paul et al. reported that the introduction of routine OCT use in a primary eyecare setting in Australia improved the rate of disease detection for glaucoma [22]. A similar improvement was observed among community optometrists in the United Kingdom (UK) for glaucoma, DR, and AMD [23]. The incorporation of a device that enables semi-automated capture of OCTA without the need of skilled operators could provide this user group with new diagnostic capabilities for detecting pathological vascular features, potentially enhancing coordinated care and screening programs. This could be particularly beneficial if OCTA becomes a standard of care for evaluating the severity of glaucoma and also, possibly, DR [8].

There are several limitations to this study. First, the management of image artifacts remains a significant factor requiring careful analysis and interpretation by users. Although various artifacts were included in the image quality grading criteria, a more comprehensive assessment of different types of artifacts could help users minimize their occurrence and enhance image capture quality. Additionally, neither device had a software version that supported quantitative metrics, which limited the ability to perform detailed comparisons of OCT metrics. While the Maestro2 has demonstrated interoperability with another OCT device (DRI OCT Triton, Topcon Corporation, Tokyo, Japan) [24], similar analyses for OCTA metrics are needed to further validate the clinical utility of the Maestro2 and support its broader adoption. Finally, limitations inherent to OCTA technology, such as its inability to assess vessel leakage, will persist in clinical practice [25].

This study demonstrates that the semi-automated Maestro2 provides OCTA scans that are comparable in clinical utility to a device that requires manual operation. This may be of particular relevance to eye-care practices or low-resource environments that are not equipped with skilled operators.

## Figures and Tables

**Table 1:** Calculation of Response Rate for Identification of Key Pathological Vascular Features between Maestro2 and Cirrus.

Maestro2 Identification	Cirrus Identification			Total
	Match	No Match	No Acceptable Scans *	
Match	n11	n12	n13	n1□
No Match	n21	n22	n23	n2□
No Acceptable Scans *	n31	n32	n33	n3□
Total	n□1	n□2	n□3	n□□

Eyes with key pathological vascular features “Cannot Grade” from FA/ICGA are excluded. Match = pathology identification (with possible outcomes of Yes, No, and Cannot Grade) is the same between OCTA and FA/ICGA. The response rate represents the total number of eyes in the shaded cells divided by the total number of eyes in the table. * Eyes that did not have acceptable scans as assessed by the study site(s). □ Expected marginal counts.

**Table 2:** Demographics and Ocular Characteristics of Study Groups.

	Normal	Pathology
Subjects (n)	38	86
Age (mean ± SD)(years)	37.9 ± 13.5	61.0 ± 15.7
Gender (M/F)	15/23	42/44
Race (n, %)		
White	28, 73.7%	61, 70.9%
African American	2, 5.3%	19, 22.1%
Other	8, 21%	7, 7%
Eyes (n)	38	86
BCVA		
20/20 or better (n, %)	35, 92.1%	17, 19.8%
Worse than 20/20	3, 7.9%	69, 80.2%
Pupil size (mean ± SD) (mm)	3.1 ± 0.9	2.8 ± 0.7
IOP (mean ± SD) (mmHg)	14.3 ± 2.2	14.7 ± 2.8

Abbreviation: BCVA, Best-corrected visual acuity; IOP, Intra-ocular pressure; SD, Standard Deviation.

**Table 3:** Proportion of Clinically Useful Images from Cirrus and Maestro2 per Image Quality Grade in Different Scan Types.

	Normal		Pathology		Total	
Scan Type	Cirrus	Maestro2	Cirrus	Maestro2	Cirrus	Maestro2
3 mm × 3 mm macula scan	86.8% (71.2%, 95.6%)	94.7% (82.3%, 99.4%)	70.9% (60.1%, 80.2%)	82.6% (72.9%, 90.0%)	75.8% (67.3%, 83.0%)	86.3% (79.0%, 91.8%)
6 mm × 6 mm macula scan	86.8% (71.2%, 95.6%)	89.5 (75.2%, 97.1%)	72.1% (61.4%, 81.2%)	82.6% (72.9%, 90.0%)	76.6% (68.2%, 83.7%)	84.7% (77.1%, 90.5%)
4.5 mm × 4.5 mm disc scan	97.4% (86.2%, 99.9%)	92.1% (78.6%, 98.3%)	72.1% (61.4%, 81.2%)	77.9% (67.7%, 86.1%)	79.8% (71.7%, 86.5%)	82.3% (74.4%, 88.5%)
95% confidence intervals are shown in parentheses						

**Table 4:** Proportion of Clinically Useful Images from Cirrus and Maestro2 for Visualizing Anatomical Vascular Features per Scan Type.

		Proportion (95% Confidence Interval)					
Scan Type	Key Vascular Features	Normal		Pathology		Total	
		Cirrus	Maestro2	Cirrus	Maestro2	Cirrus	Maestro2
3 mm × 3 mm Macula Scan	Foveal Avascular Zone	86.8% (71.2%, 95.6%)	94.7% (82.3%, 99.4%)	62.8% (51.7%, 73.0%)	77.9% (67.7%, 86.1%)	70.2% (61.3%, 78.0%)	83.1% (75.3%, 89.2%)
	Medium Blood Vessels	86.8% (71.2%, 95.6%)	94.7% (82.3%, 99.4%)	72.1% (61.4%, 81.2%)	84.9% (75.6%, 91.7%)	76.6% (68.2%, 83.7%)	87.9% (80.8%, 93.1%)
	Small Blood Vessels	86.8% (71.2%, 95.6%)	94.7% (82.3%, 99.4%)	72.1% (61.4%, 81.2%)	83.7% (74.2%, 90.8%)	76.6% (68.2%, 83.7%)	87.1% (80.0%, 92.4%)
6 mm × 6 mm Macula Scan	Foveal Avascular Zone	89.5% (75.2%, 97.1%)	89.5% (75.2%, 97.1%)	70.9% (60.1%, 80.2%)	81.4% (71.6%, 89.0%)	76.6% (68.2%, 83.7%)	83.9% (76.2%, 89.9%)
	Large Blood Vessels	89.5% (75.2%, 97.1%)	92.1% (78.6%, 98.3%)	73.3% (62.6%, 82.2%)	86.1% (76.9%, 92.6%)	78.2% (69.9%, 85.1%)	87.9% (80.8%, 93.1%)
	Medium Blood Vessels	89.5% (75.2%, 97.1%)	92.1% (78.6%, 98.3%)	73.3% (62.6%, 82.2%)	86.1% (76.9%, 92.6%)	78.2% (69.9%, 85.1%)	87.9% (80.8%, 93.1%)
	Small Blood Vessels	86.8% (71.2%, 95.6%)	92.1% (78.6%, 98.3%)	73.3% (62.6%, 82.2%)	86.1% (76.9%, 92.6%)	77.4% (69.0%, 84.4%)	87.9% (80.8%, 93.1%)
4.5 mm × 4.5 mm Disc Scan	Large Blood Vessels	97.4% (86.2%, 99.9%)	92.1% (78.6%, 98.3%)	75.6% (65.1%, 84.2%)	80.2% (70.3%, 88.0%)	82.3% (74.4%, 88.5%)	83.9% (76.2%, 89.9%)
	Medium Blood Vessels	97.4% (86.2%, 99.9%)	92.1% (78.6%, 98.3%)	75.6% (65.1%, 84.2%)	79.1% (69.0%, 87.1%)	82.3% (74.4%, 88.5%)	83.1% (75.3%, 89.2%)
	Small Blood Vessels	97.4% (86.2%, 99.9%)	92.1% (78.6%, 98.3%)	75.6% (65.1%, 84.2%)	79.1% (69.0%, 87.1%)	82.3% (74.4%, 88.5%)	83.1% (75.3%, 89.2%)

**Table 5:** Consensus OCTA Identification of Key Pathological Vascular Feature per FA/ICGA.

Scan Type	Key Pathological Vascular Feature	PPA		NPA	
		Cirrus	Maestro2	Cirrus	Maestro2
Normal					
3 mm × 3 mm Macula Scan	MAs	0/1 (0.0%)	0/2 (0.0%)	32/32 (100%)	34/34 (100%)
	RI/CD	0/0 (--%)	0/0 (--%)	30/33 (90.9%)	35/36 (97.2%)
	CNV	1/1 (100%)	1/1 (100%)	32/32 (100%)	35/35 (100%)
	PPOI	2/2 (100%)	2/3 (66.7%)	29/31 (93.5%)	33/33 (100%)
6 mm × 6 mm Macula Scan	MAs	0/2 (0.0%)	0/2 (0.0%)	31/32 (96.9%)	33/33 (100%)
	RI/CD	0/0 (--%)	0/0 (--%)	33/34 (97.1%)	33/35 (94.3%)
	CNV	1/1 (100%)	1/1 (100%)	33/33 (100%)	34/34 (100%)
	PPOI	1/3 (33.3%)	1/3 (33.3%)	30/31 (96.8%)	31/32 (96.9%)
4.5 mm × 4.5 mm Disc Scan	MAs	0/1 (0.0%)	0/1 (0.0%)	36/36 (100%)	34/34 (100%)
	RI/CD	0/0 (--%)	0/0 (--%)	35/37 (94.6%)	35/35 (100%)
	CNV	0/0 (--%)	0/0 (--%)	37/37 (100%)	35/35 (100%)
	PPOI	0/1 (0.0%)	0/1 (0.0%)	34/36 (94.4%)	34/34 (100%)
Pathology					
3 mm × 3 mm Macula Scan	MAs	25/26 (96.2%)	28/29 (96.6%)	31/33 (93.9%)	38/41 (92.7%)
	RI/CD	27/27 (100%)	27/29 (93.1%)	27/32 (84.4%)	35/41 (85.4%)
	CNV	13/15 (86.7%)	16/18 (88.9%)	38/44 (86.4%)	43/52 (82.7%)
	PPOI	24/25 (96.0%)	30/31 (96.8%)	26/34 (76.5%)	27/39 (69.2%)
6 mm × 6 mm Macula Scan	MAs	23/24 (95.8%)	28/29 (96.6%)	33/35 (94.3%)	38/41 (92.7%)
	RI/CD	24/25 (96.0%)	29/29 (100%)	31/34 (91.2%)	36/41 (87.8%)
	CNV	14/17 (82.4%)	16/19 (84.2%)	36/42 (85.7%)	43/51 (84.3%)
	PPOI	25/28 (89.3%)	30/32 (93.8%)	21/31 (67.7%)	27/38 (71.1%)
4.5 mm × 4.5 mm Disc Scan	MAs	14/20 (70.0%)	17/23 (73.9%)	40/42 (95.2%)	44/44 (100%)
	RI/CD	17/21 (81.0%)	18/24 (75.0%)	30/40 (75.0%)	36/42 (85.7%)
	CNV	2/2 (100%)	2/3 (66.7%)	59/59 (100%)	62/63 (98.4%)
	PPOI	10/17 (58.8%)	14/18 (77.8%)	42/44 (95.5%)	46/48 (95.8%)
Total					
3 mm × 3 mm Macula Scan	MAs	25/27 (92.6%)	28/31 (90.3%)	63/65 (96.9%)	72/75 (96.0%)
	RI/CD	27/27 (100%)	27/29 (93.1%)	57/65 (87.7%)	70/77 (90.9%)
	CNV	14/16 (87.5%)	17/19 (89.5%)	70/76 (92.1%)	78/87 (89.7%)
	PPOI	26/27 (96.3%)	32/34 (94.1%)	55/65 (84.6%)	60/72 (83.3%)
6 mm × 6 mm Macula Scan	MAs	23/26 (88.5%)	28/31 (90.3%)	64/67 (95.5%)	71/74 (95.9%)
	RI/CD	24/25 (96.0%)	29/29 (100%)	64/68 (94.1%)	69/76 (90.8%)
	CNV	15/18 (83.3%)	17/20 (85.0%)	69/75 (92.0%)	77/85 (90.6%)
	PPOI	26/31 (83.9%)	31/35 (88.6%)	51/62 (82.3%)	58/70 (82.9%)
4.5 mm × 4.5 mm Disc Scan	MAs	14/21 (66.7%)	17/24 (70.8%)	76/78 (97.4%)	78/78 (100%)
	RI/CD	17/21 (81.0%)	18/24 (75.0%)	65/77 (84.4%)	71/77 (92.2%)
	CNV	2/2 (100%)	2/3 (66.7%)	96/96 (100%)	97/98 (99.0%)
	PPOI	10/18 (55.6%)	14/19 (73.7%)	76/80 (95.0%)	80/82 (97.6%)

Abbreviations: PPA, positive percent agreement, NPA, negative percent agreement, MAs, microaneurysms, RI/CD, retinal ischemia/capillary dropout, CNV, choroidal neovascularization, PPOI, primary pathology of interest.

**Table 6:** Response Rate Summary of Rate of Matched OCTA and FA/ICGA on Consensus Identification of Key Vascular Pathologies per Scan Type.

Scan Type	Key Vascular Pathologies	Response Rate (95% Confidence Interval)		
		Normal	Pathology	Total
3 mm × 3 mm Macula Scan	MAs	92.1% (78.6%, 98.3%)	81.0% (70.9%, 88.7%)	84.4% (76.8%, 90.4%)
	RI/CD	94.7% (82.3%, 99.4%)	76.2% (65.7%, 84.8%)	82.0% (74.0%, 88.3%)
	CNV	94.7% (82.3%, 99.4%)	79.8% (69.6%, 87.7%)	84.4% (76.8%, 90.4%)
	PPOI	92.1% (78.6%, 98.3%)	77.4% (67.0%, 85.8%)	82.0% (74.0%, 88.3%)
6 mm × 6 mm Macula Scan	MAs	92.1% (78.6%, 98.3%)	82.1% (72.3%, 89.6%)	85.2% (77.7%, 91.0%)
	RI/CD	89.5% (75.2%, 97.1%)	79.8% (69.6%, 87.7%)	82.8% (74.9%, 89.0%)
	CNV	92.1% (78.6%, 98.3%)	81.0% (70.9%, 88.7%)	84.4% (76.8%, 90.4%)
	PPOI	92.1% (78.6%, 98.3%)	78.6% (68.3%, 86.8%)	82.8% (74.9%, 89.0%)
4.5 mm × 4.5 mm Disc Scan	MAs	92.1% (78.6%, 98.3%)	75.6% (65.1%, 84.2%)	80.6% (72.6%, 87.2%)
	RI/CD	92.1% (78.6%, 98.3%)	71.8% (61.0%, 81.0%)	78.0% (69.7%, 85.0%)
	CNV	92.1% (78.6%, 98.3%)	75.3% (64.7%, 84.0%)	80.5% (72.4%, 87.1%)
	PPOI	92.1% (78.6%, 98.3%)	75.3% (64.7%, 84.0%)	80.5% (72.4%, 87.1%)

Abbreviations: MAs, microaneurysms, RI/CD, retinal ischemia/capillary dropout, CNV, choroidal neovascularization, PPOI, primary pathology of interest.

## Data Availability

Dataset available on request from the authors.

## Supplementary Materials

The following supporting information can be downloaded at: https://www.mdpi.com/article/10.3390/jcm13216301/s1, Table S1: Inter-grader Agreement in OCTA Image Quality; Table S2: Inter-grader Agreement in Visualizing Anatomical Vascular Features on OCTA Images; Table S3: Identification of Key Pathological Vascular Features by Consensus OCTA per FA/ICGA; Table S4: Inter-grader Agreement in Identification of Key Pathological Vascular Feature on OCTA Images.

## Appendix A

Image Grading Criteria

- Image quality

OCTA image quality grading was based on a scale of poor, average, or good. One scan per scan type was graded per study eye per device. All three graders graded the images independently, and final scores were provided based on consensus for each scan.

OCTA image quality grading took the following factors into account:

1. Low overall signal strength
2. Poor illumination/regional weak signal
3. Poor focus/blurry image
4. Motion artifacts
5. Clipping
6. Blinking
7. Unexpected projection artifacts preventing vascular feature visualization

Each OCTA scan received an image quality score per grader, with the final quality score determined based on grader consensus.

A guideline for image quality grading was provided as follows:

For an OCTA scan to be graded as “Good”, the following criteria must be fulfilled:

1. The image is mostly free of all image quality issues, and
2. All key anatomical vascular features expected within the scan area are visualized and the presence/absence of all key pathological vascular features can be assessed.

For an OCTA scan to be graded as “Average”, the following criteria must be fulfilled:

1. Some parts of the image are affected by one or more image quality issues, and
2. All key anatomical vascular features expected within the scan area are at least partially visualized or the presence/absence of at least one of the key pathological vascular features can be graded.

For an OCTA scan to be graded as “Poor”, the following criteria must be fulfilled:

1. Large parts of the image are affected by one or more image quality issues, and
2. At least one of the key anatomical vascular features expected within the scan area is not visible or the presence/absence of all key pathological vascular features cannot be graded due to image quality issues.

- Visibility of anatomical vascular features

Visibility of the anatomical vascular features was graded based on scale of “Not Visible”, “Partially Visible”, and “Visible”. It should be noted that a vascular structure may not have been gradable due to the location of the OCTA scan, a grade of “NA” or “Not Applicable” was assigned. One scan per scan type per study eye per device was graded. All three graders graded the images independently. The final scores were based on consensus as described above.

The following anatomical vascular features were graded:

1. Foveal avascular zone (FAZ) border (“NA” if not fully included in the scan field, such as a 3 mm × 3 mm macular scan acquired away from the fovea; FAZ was graded “NA” for all 4.5 mm × 4.5 mm disc scans)
2. Large-sized blood vessels (large-sized blood vessels was graded “NA” for all 3 mm × 3 mm macular scans)
3. Medium-sized blood vessels
4. Small blood vessels/capillaries

Each OCTA scan received a score per feature per grader, with the final quality score per feature determined based on grader consensus.

1. FAZ: If the entire FAZ margin was visualized, the image was graded as “Visible”. If only some sections of the FAZ margin were visualized, the image was graded as “Partially Visible”. If the FAZ margin was mostly not visualized, the image was graded as “Not Visible”. “Not Applicable” should be selected if the scan area did not capture the FAZ or if it was a 4.5 mm × 4.5 mm disc scan.
2. Large blood vessels: If the large blood vessels were expected to lie within the scan area and the pattern was clearly visualized, the image was graded as “Visible”. If some large blood vessels were visualized and the pattern was clear in parts of the image, the image was graded as “Partially Visible”. If the large blood vessels and the pattern were mostly not visualized, the image was graded as “Not Visible”. “Not Applicable” should be selected if the scan area did not capture the large blood vessels.
3. Medium blood vessels: If the medium blood vessels were expected to lie within the scan area and the pattern was clearly visualized, the image was graded as “Visible”. If some medium blood vessels were visualized and the pattern was clear in parts of the image, the image was graded as “Partially Visible”. If the medium blood vessels and the pattern were mostly not visualized, the image was graded as “Not Visible”. “Not Applicable” should be selected if the scan area did not capture the medium blood vessels.
4. Small blood vessels: If the small blood vessels/capillaries expected to lie within the scan area were all visualized, the image was graded as “Visible”. If some small blood vessels/capillaries were visualized, the image was graded as “Partially Visible”. If the small blood vessels/capillaries were mostly not visualized, the image was graded as “Not Visible”. “Not Applicable” should be selected if the scan area did not capture the small blood vessels/capillaries.

- Identification of Pathological Vascular Features

Presence/absence of pathology was graded for OCTA scans and FA/ICGA images within each of the boxes demarcating OCTA scan areas, respectively. Identification of the key pathological vascular features was graded based on a scale of “Cannot Grade” for unable to distinguish the presence/absence of a pathological vascular feature, “No” for a pathological vascular feature not observed, and “Yes” for a pathological vascular feature observed.

The following key pathological vascular features were graded respectively for each study eye and for each imaging modality (Maestro2 OCTA, Cirrus OCTA, and FA/ICGA)

1. microaneurysms (MAs)
2. retinal ischemia/capillary dropout/(RI/CD)
3. choroidal neovascularization (CNV)

Other vascular features, such as macular telangiectasias (MacTel) and retinal neovascularization (RNV), etc., were noted in grading form, if observed.

Each OCTA device received a score per feature per grader, per scan type, per study eye, with the final quality score determined based on grader consensus.

Each set of FA/ICGA of a study eye received a score per feature, per grader, per boxed area, with the final quality score determined based on grader consensus.

1. MAs: If MAs were present, the image was graded as “Yes” (the grader should have been at least 50% certain). If MAs were not present, the image was graded as “No” (the grader should have been at least 50% certain). If the grader was unable to assess the presence or absence of MAs within the OCTA scan, the image was graded as “Cannot Grade”.
2. RI/CD: If RI/CD was present, the image was graded as “Yes” (the grader should have been at least 50% certain). If RI/CD was not present, the image was graded as “No” (the grader should have been at least 50% certain). If the grader was unable to assess the presence or absence of RI/CD within the OCTA scan, the image was graded as “Cannot Grade”.
3. CNV: If CNV was present, the image was graded as “Yes” (the grader should have been at least 50% certain). If CNV was not present, the image was graded as “No” (the grader should have been at least 50% certain). If the grader was unable to assess the presence or absence of CNV within the OCTA scan, the image was graded as “Cannot Grade”.
