# Supplementary material for: Clinical Performance of Semi-Automated Spectral-Domain Optical Coherence Tomography Angiography
Source: J Clin Med. 2024 Oct 22;13(21):6301. doi: 10.3390/jcm13216301 (PMC11546602; doi:10.3390/jcm13216301)
Supplement: Supplementary file 1 [file jcm-13-06301-s001.zip › jcm-3218421-supplementary.pdf]

Supplementary Materials: Table S1: Inter-grader Agreement in OCTA Image Quality; Table S2: Inter-grader Agreement in Visualizing Anatomical Vascular Features on OCTA Images; Table S3: Identification of Key Pathological Vascular Features by Consensus OCTA per FA/ICGA; Table S4: Inter-grader Agreement in Identification of Key Pathological Vascular Feature on OCTA Images.

Table S1. Inter-grader Agreement in OCTA Image Quality

**Device**	**Scan Type**	**Agreement % (95% Confidence Interval)**	**Normal**	**Pathology**	**Total**
Cirrus	3mm×3mm Macula	Same grade from 3 graders	39.4% (22.9%, 57.9%)	14.5% (6.9%, 25.8%)	23.2% (15.1%, 32.9%)
		Same grade from 2 graders	60.6% (42.1%, 77.1%)	85.5% (74.2%, 93.1%)	76.8% (67.1%, 84.9%)
		Different grades from 3 graders	0.0%	0.0%	0.0%
	6mm×6mm Macula	Same grade from 3 graders	29.4% (15.1%, 47.5%)	28.6% (17.9%, 41.3%)	28.9% (20.1%, 39.0%)
		Same grade from 2 graders	70.6% (52.5%, 84.9%)	71.4% (58.7%, 82.1%)	71.1% (61.1%, 80.0%)
		Different grades from 3 graders	0.0%	0.0%	0.0%
	4.5mm×4.5mm Disc	Same grade from 3 graders	32.4% (18.0%, 49.8%)	24.6% (14.8%, 36.9%)	27.5% (19.1%, 37.2%)
		Same grade from 2 graders	67.6% (50.2%, 82.0%)	75.4% (63.1%, 85.2%)	72.6% (62.8%, 80.9%)
		Different grades from 3 graders	0.0%	0.0%	0.0%
Maestro2	3mm×3mm Macula	Same grade from 3 graders	30.6% (16.3%, 48.1%)	19.2% (10.9%, 30.1%)	22.9% (15.4%, 32.0%)
		Same grade from 2 graders	69.4% (51.9%, 83.7%)	80.8% (69.9%, 89.1%)	77.1% (68.0%, 84.6%)
		Different grades from 3 graders	0.0%	0.0%	0.0%
	6mm×6mm Macula	Same grade from 3 graders	8.6% (1.8%, 23.1%)	27.0% (17.4%, 38.6%)	21.1% (13.9%, 30.0%)
		Same grade from 2 graders	91.4% (76.9%, 98.2%)	73.0% (61.4%, 82.6%)	78.9% (70.0%, 86.1%)
		Different grades from 3 graders	0.0%	0.0%	0.0%
	4.5mm×4.5mm Disc	Same grade from 3 graders	8.6% (1.8%, 23.1%)	14.5% (7.2%, 25.0%)	12.5% (6.8%, 20.4%)
		Same grade from 2 graders	91.4% (76.9%, 98.2%)	85.5% (75.0%, 92.8%)	87.5% (79.6%, 93.2%)
		Different grades from 3 graders	0.0%	0.0%	0.0%

Table S2. Inter-grader Agreement in Visualizing Anatomical Vascular Features on OCTA Images

		**Agreement % (95% Confidence Interval)**		
		**Normal**	**Pathology**	**Total**
**Cirrus 3mm×3mm Macula Scan**				
**Foveal Avascular Zone**	Same grade from 3 graders	66.7% (48.2%, 82.0%)	50.9% (37.1%, 64.6%)	56.8% (45.8%, 67.3%)
	Same grade from 2 graders	33.3% (18.0%, 51.8%)	49.1% (35.4%, 62.9%)	43.2% (32.7%, 54.2%)
	Different grades from 3 graders	0.0%	0.0%	0.0%
**Medium Blood Vessels**	Same grade from 3 graders	63.6% (45.1%, 79.6%)	35.5% (23.7%, 48.7%)	45.3% (35.0%, 55.8%)

	Same grade from 2 graders	36.4% (20.4%, 54.9%)	64.5% (51.3%, 76.3%)	54.7% (44.2%, 65.0%)
	Different grades from 3 graders	0.0%	0.0%	0.0%
**Small Blood Vessels**	Same grade from 3 graders	57.6% (39.2%, 74.5%)	17.7% (9.2%, 29.5%)	31.6% (22.4%, 41.9%)
	Same grade from 2 graders	42.4% (25.5%, 60.8%)	82.3% (70.5%, 90.8%)	68.4% (58.1%, 77.6%)
	Different grades from 3 graders	0.0%	0.0%	0.0%
**Cirrus 6mm×6mm Macula Scan**				
**Foveal Avascular Zone**	Same grade from 3 graders	70.6% (52.5%, 84.9%)	32.8% (21.3%, 46.0%)	46.3% (36.0%, 56.8%)
	Same grade from 2 graders	29.4% (15.1%, 47.5%)	67.2% (54.0%, 78.7%)	53.7% (43.2%, 64.0%)
	Different grades from 3 graders	0.0%	0.0%	0.0%
**Large Blood Vessels**	Same grade from 3 graders	91.2% (76.3%, 98.1%)	73.0% (60.3%, 83.4%)	79.4% (70.0%, 86.9%)
	Same grade from 2 graders	8.8% (1.9%, 23.7%)	27.0% (16.6%, 40.0%)	20.6% (13.1%, 30.0%)
	Different grades from 3 graders	0.0%	0.0%	0.0%
**Medium Blood Vessels**	Same grade from 3 graders	44.1% (27.2%, 62.1%)	20.6% (11.5%, 32.7%)	28.9% (20.1%, 39.0%)
	Same grade from 2 graders	55.9% (37.9%, 72.8%)	79.4% (67.3%, 88.5%)	71.1% (61.1%, 79.9%)
	Different grades from 3 graders	0.0%	0.0%	0.0%
**Small Blood Vessels**	Same grade from 3 graders	29.4% (15.1%, 47.5%)	17.5% (9.1%, 29.1%)	21.6% (13.9%, 31.2%)
	Same grade from 2 graders	70.6% (52.5%, 84.9%)	82.5% (70.9%, 91.0%)	78.4% (68.8%, 86.1%)
	Different grades from 3 graders	0.0%	0.0%	0.0%
**Cirrus 4.5mm×4.5mm Disc Scan**				
**Large Blood Vessels**	Same grade from 3 graders	64.9% (47.5%, 79.8%)	64.6% (51.8%, 76.1%)	64.7% (54.6%, 73.9%)
	Same grade from 2 graders	35.1% (20.2%, 52.5%)	35.4% (23.9%, 48.2%)	35.3% (26.1%, 45.4%)
	Different grades from 3 graders	0.0%	0.0%	0.0%
**Medium Blood Vessels**	Same grade from 3 graders	54.1% (36.9%, 70.5%)	30.8% (19.9%, 43.4%)	39.2% (29.7%, 49.4%)
	Same grade from 2 graders	45.9% (29.5%, 63.1%)	69.2% (56.6%, 80.0%)	60.8% (50.6%, 70.3%)
	Different grades from 3 graders	0.0%	0.0%	0.0%
**Small Blood Vessels**	Same grade from 3 graders	45.9% (29.5%, 63.1%)	26.2% (16.0%, 38.5%)	33.3% (24.3%, 43.4%)
	Same grade from 2 graders	54.1% (36.9%, 70.5%)	73.8% (61.5%, 84.0%)	66.7% (56.6%, 75.7%)
	Different grades from 3 graders	0.0%	0.0%	0.0%
**Maestro2 3mm×3mm Macula Scan**				
**Foveal Avascular Zone**	Same grade from 3 graders	58.3% (40.8%, 74.5%)	37.7% (26.3%, 50.2%)	44.8% (35.0%, 54.8%)
	Same grade from 2 graders	41.7% (25.5%, 59.2%)	62.3% (49.8%, 73.7%)	55.2% (45.2%, 65.0%)
	Different grades from 3 graders	0.0%	0.0%	0.0%
**Medium Blood Vessels**	Same grade from 3 graders	38.9% (23.1%, 56.5%)	20.5% (12.0%, 31.6%)	26.6% (18.6%, 35.9%)
	Same grade from 2 graders	61.1% (43.5%, 76.9%)	79.5% (68.4%, 88.0%)	73.4% (64.1%, 81.4%)
	Different grades from 3 graders	0.0%	0.0%	0.0%
**Small Blood Vessels**	Same grade from 3 graders	25.0% (12.1%, 42.2%)	11.0% (4.9%, 20.5%)	15.6% (9.4%, 23.8%)
	Same grade from 2 graders	75.0% (57.8%, 87.9%)	89.0% (79.5%, 95.2%)	84.4% (76.2%, 90.6%)
	Different grades from 3 graders	0.0%	0.0%	0.0%
**Maestro2 6mm×6mm Macula Scan**				
**Foveal Avascular Zone**	Same grade from 3 graders	45.7% (28.8%, 63.4%)	32.4% (21.8%, 44.5%)	36.8% (27.6%, 46.7%)
	Same grade from 2 graders	54.3% (36.7%, 71.2%)	67.6% (55.5%, 78.2%)	63.2% (53.3%, 72.4%)
	Different grades from 3 graders	0.0%	0.0%	0.0%
**Large Blood Vessels**	Same grade from 3 graders	74.3% (56.7%, 87.5%)	48.6% (36.9%, 60.6%)	56.9% (47.0%, 66.3%)
	Same grade from 2 graders	25.7% (12.5%, 43.3%)	51.4% (39.4%, 63.2%)	43.1% (33.7%, 53.0%)

	Different grades from 3 graders	0.0%	0.0%	0.0%
**Medium Blood Vessels**	Same grade from 3 graders	28.6% (14.6%, 46.3%)	20.3% (11.8%, 31.2%)	22.9% (15.4%, 32.0%)
	Same grade from 2 graders	71.4% (53.7%, 85.4%)	79.7% (68.8%, 88.2%)	77.1% (68.0%, 84.6%)
	Different grades from 3 graders	0.0%	0.0%	0.0%
**Small Blood Vessels**	Same grade from 3 graders	17.1% (6.6%, 33.6%)	18.9% (10.7%, 29.7%)	18.3% (11.6%, 26.9%)
	Same grade from 2 graders	82.9% (66.4%, 93.4%)	81.1% (70.3%, 89.3%)	81.7% (73.1%, 88.4%)
	Different grades from 3 graders	0.0%	0.0%	0.0%
**Maestro2 4.5mm×4.5mm Disc Scan**				
**Large Blood Vessels**	Same grade from 3 graders	62.9% (44.9%, 78.5%)	50.7% (38.4%, 63.0%)	54.8% (44.7%, 64.6%)
	Same grade from 2 graders	37.1% (21.5%, 55.1%)	49.3% (37.0%, 61.6%)	45.2% (35.4%, 55.3%)
	Different grades from 3 graders	0.0%	0.0%	0.0%
**Medium Blood Vessels**	Same grade from 3 graders	31.4% (16.9%, 49.3%)	11.6% (5.1%, 21.6%)	18.3% (11.4%, 27.1%)
	Same grade from 2 graders	68.6% (50.7%, 83.2%)	88.4% (78.4%, 94.9%)	81.7% (73.0%, 88.6%)
	Different grades from 3 graders	0.0%	0.0%	0.0%
**Small Blood Vessels**	Same grade from 3 graders	11.4% (3.2%, 26.7%)	14.5% (7.2%, 25.0%)	13.5% (7.6%, 21.6%)
	Same grade from 2 graders	88.6% (73.3%, 96.8%)	85.5% (75.0%, 92.8%)	86.5% (78.5%, 92.4%)
	Different grades from 3 graders	0.0%	0.0%	0.0%

FAZ Border was Not Applicable (NA, not graded) for the 4.5mm×4.5mm Disc scans; Large Blood Vessels was NA for the 3mm×3mm Macula scans. These images were not included in the visualization analysis.

Table S3. Identification of Key Pathological Vascular Features by Consensus OCTA per FA/ICGA

Scan Type	Key Pathological Vascular Feature	PPA (95% confidence interval)		NPA (95% confidence interval)	
		Cirrus	Maestro2	Cirrus	Maestro2
**Normal**					
3mm×3mm Macula Scan	MAs	0/1 (0.0%) (0.0%, 97.5%)	0/2 (0.0%) (0.0%, 84.2%)	32/32 (100%) (89.1%, 100%)	34/34 (100%) (89.7%, 100%)
	RI/CD	0/0 (--%)	0/0 (--%)	30/33 (90.9%) (75.7%, 98.1%)	35/36 (97.2%) (85.5%, 99.9%)
	CNV	1/1 (100%) (2.5%, 100%)	1/1 (100%) (2.5%, 100%)	32/32 (100%) (89.1%, 100%)	35/35 (100%) (90.0%, 100%)
	PPOI	2/2 (100%) (15.8%, 100%)	2/3 (66.7%) (9.4%, 99.2%)	29/31 (93.5%) (78.6%, 99.2%)	33/33 (100%) (89.4%, 100%)
6mm×6mm Macula Scan	MAs	0/2 (0.0%) (0.0%, 84.2%)	0/2 (0.0%) (0.0%, 84.2%)	31/32 (96.9%) (83.8%, 99.9%)	33/33 (100%) (89.4%, 100%)
	RI/CD	0/0 (--%)	0/0 (--%)	33/34 (97.1%) (84.7%, 99.9%)	33/35 (94.3%) (80.8%, 99.3%)
	CNV	1/1 (100%) (2.5%, 100%)	1/1 (100%) (2.5%, 100%)	33/33 (100%) (89.4%, 100%)	34/34 (100%) (89.7%, 100%)
	PPOI	1/3 (33.3%) (0.8%, 90.6%)	1/3 (33.3%) (0.8%, 90.6%)	30/31 (96.8%) (83.3%, 99.9%)	31/32 (96.9%) (83.8%, 99.9%)
4.5mm×4.5mm Disc Scan	MAs	0/1 (0.0%) (0.0%, 97.5%)	0/1 (0.0%) (0.0%, 97.5%)	36/36 (100%) (90.3%, 100%)	34/34 (100%) (89.7%, 100%)
	RI/CD	0/0 (--%)	0/0 (--%)	35/37 (94.6%) (81.8%, 99.3%)	35/35 (100%) (90.0%, 100%)
	CNV	0/0 (--%)	0/0 (--%)	37/37 (100%) (90.5%, 100%)	35/35 (100%) (90.0%, 100%)
	PPOI	0/1 (0.0%)	0/1 (0.0%)	34/36 (94.4%)	34/34 (100%)

		(0.0%, 97.5%)	(0.0%, 97.5%)	(81.3%, 99.3%)	(89.7%, 100%)
**Pathology**					
3mm×3mm Macula Scan	MAs	25/26 (96.2%)<br>(80.4%, 99.9%)	28/29 (96.6%)<br>(82.2%, 99.9%)	31/33 (93.9%)<br>(79.8%, 99.3%)	38/41 (92.7%)<br>(80.1%, 98.5%)
	RI/CD	27/27 (100%)<br>(87.2%, 100%)	27/29 (93.1%)<br>(77.2%, 99.2%)	27/32 (84.4%)<br>(67.2%, 94.7%)	35/41 (85.4%)<br>(70.8%, 94.4%)
	CNV	13/15 (86.7%)<br>(59.5%, 98.3%)	16/18 (88.9%)<br>(65.3%, 98.6%)	38/44 (86.4%)<br>(72.6%, 94.8%)	43/52 (82.7%)<br>(69.7%, 91.8%)
	PPOI	24/25 (96.0%)<br>(79.6%, 99.9%)	30/31 (96.8%)<br>(83.3%, 99.9%)	26/34 (76.5%)<br>(58.8%, 89.3%)	27/39 (69.2%)<br>(52.4%, 83.0%)
6mm×6mm Macula Scan	MAs	23/24 (95.8%)<br>(78.9%, 99.9%)	28/29 (96.6%)<br>(82.2%, 99.9%)	33/35 (94.3%)<br>(80.8%, 99.3%)	38/41 (92.7%)<br>(80.1%, 98.5%)
	RI/CD	24/25 (96.0%)<br>(79.6%, 99.9%)	29/29 (100%)<br>(88.1%, 100%)	31/34 (91.2%)<br>(76.3%, 98.1%)	36/41 (87.8%)<br>(73.8%, 95.9%)
	CNV	14/17 (82.4%)<br>(56.6%, 96.2%)	16/19 (84.2%)<br>(60.4%, 96.6%)	36/42 (85.7%)<br>(71.5%, 94.6%)	43/51 (84.3%)<br>(71.4%, 93.0%)
	PPOI	25/28 (89.3%)<br>(71.8%, 97.7%)	30/32 (93.8%)<br>(79.2%, 99.2%)	21/31 (67.7%)<br>(48.6%, 83.3%)	27/38 (71.1%)<br>(54.1%, 84.6%)
4.5mm×4.5mm Disc Scan	MAs	14/20 (70.0%)<br>(45.7%, 88.1%)	17/23 (73.9%)<br>(51.6%, 89.8%)	40/42 (95.2%)<br>(83.8%, 99.4%)	44/44 (100%)<br>(92.0%, 100%)
	RI/CD	17/21 (81.0%)<br>(58.1%, 94.6%)	18/24 (75.0%)<br>(53.3%, 90.2%)	30/40 (75.0%)<br>(58.8%, 87.3%)	36/42 (85.7%)<br>(71.5%, 94.6%)
	CNV	2/2 (100%)<br>(15.8%, 100%)	2/3 (66.7%)<br>(9.4%, 99.2%)	59/59 (100%)<br>(93.9%, 100%)	62/63 (98.4%)<br>(91.5%, 100%)
	PPOI	10/17 (58.8%)<br>(32.9%, 81.6%)	14/18 (77.8%)<br>(52.4%, 93.6%)	42/44 (95.5%)<br>(84.5%, 99.4%)	46/48 (95.8%)<br>(85.7%, 99.5%)
**Total**					
3mm×3mm Macula Scan	MAs	25/27 (92.6%)<br>(75.7%, 99.1%)	28/31 (90.3%)<br>(74.2%, 98.0%)	63/65 (96.9%)<br>(89.3%, 99.6%)	72/75 (96.0%)<br>(88.8%, 99.2%)
	RI/CD	27/27 (100%)<br>(87.2%, 100%)	27/29 (93.1%)<br>(77.2%, 99.2%)	57/65 (87.7%)<br>(77.2%, 94.5%)	70/77 (90.9%)<br>(82.2%, 96.3%)
	CNV	14/16 (87.5%)<br>(61.7%, 98.4%)	17/19 (89.5%)<br>(66.9%, 98.7%)	70/76 (92.1%)<br>(83.6%, 97.0%)	78/87 (89.7%)<br>(81.3%, 95.2%)
	PPOI	26/27 (96.3%)<br>(81.0%, 99.9%)	32/34 (94.1%)<br>(80.3%, 99.3%)	55/65 (84.6%)<br>(73.5%, 92.4%)	60/72 (83.3%)<br>(72.7%, 91.1%)
6mm×6mm Macula Scan	MAs	23/26 (88.5%)<br>(69.8%, 97.6%)	28/31 (90.3%)<br>(74.2%, 98.0%)	64/67 (95.5%)<br>(87.5%, 99.1%)	71/74 (95.9%)<br>(88.6%, 99.2%)
	RI/CD	24/25 (96.0%)<br>(79.6%, 99.9%)	29/29 (100%)<br>(88.1%, 100%)	64/68 (94.1%)<br>(85.6%, 98.4%)	69/76 (90.8%)<br>(81.9%, 96.2%)
	CNV	15/18 (83.3%)<br>(58.6%, 96.4%)	17/20 (85.0%)<br>(62.1%, 96.8%)	69/75 (92.0%)<br>(83.4%, 97.0%)	77/85 (90.6%)<br>(82.3%, 95.8%)
	PPOI	26/31 (83.9%)<br>(66.3%, 94.5%)	31/35 (88.6%)<br>(73.3%, 96.8%)	51/62 (82.3%)<br>(70.5%, 90.8%)	58/70 (82.9%)<br>(72.0%, 90.8%)
4.5mm×4.5mm Disc Scan	MAs	14/21 (66.7%)<br>(43.0%, 85.4%)	17/24 (70.8%)<br>(48.9%, 87.4%)	76/78 (97.4%)<br>(91.0%, 99.7%)	78/78 (100%)<br>(95.4%, 100%)
	RI/CD	17/21 (81.0%)<br>(58.1%, 94.6%)	18/24 (75.0%)<br>(53.3%, 90.2%)	65/77 (84.4%)<br>(74.4%, 91.7%)	71/77 (92.2%)<br>(83.8%, 97.1%)
	CNV	2/2 (100%)<br>(15.8%, 100%)	2/3 (66.7%)<br>(9.4%, 99.2%)	96/96 (100%)<br>(96.2%, 100%)	97/98 (99.0%)<br>(94.4%, 100%)
	PPOI	10/18 (55.6%)<br>(30.8%, 78.5%)	14/19 (73.7%)<br>(48.8%, 90.9%)	76/80 (95.0%)<br>(87.7%, 98.6%)	80/82 (97.6%)<br>(91.5%, 99.7%)

Table S4. Inter-grader Agreement in Identification of Key Pathological Vascular Feature on OCTA Images

Key Pathological Vascular Features		Agreement % (95% Confidence Interval)		
		Normal	Pathology	Total
**Cirrus 3mm×3mm Macula Scan**				
**MAs**	Same grade from 3 graders	97.0% (84.2%, 99.9%)	90.3% (80.1%, 96.4%)	92.6% (85.4%, 97.0%)
	Same grade from 2 graders	3.0% (0.1%, 15.8%)	9.7% (3.6%, 19.9%)	7.4% (3.0%, 14.6%)
	Different grades from 3 graders	0.0%	0.0%	0.0%
**RI/CD**	Same grade from 3 graders	90.9% (75.7%, 98.1%)	82.3% (70.5%, 90.8%)	85.3% (76.5%, 91.7%)
	Same grade from 2 graders	9.1% (1.9%, 24.3%)	17.7% (9.2%, 29.5%)	14.7% (8.3%, 23.5%)
	Different grades from 3 graders	0.0%	0.0%	0.0%
**CNV**	Same grade from 3 graders	100% (89.4%, 100%)	93.5% (84.3%, 98.2%)	95.8% (89.6%, 98.8%)
	Same grade from 2 graders	0.0%	6.5% (1.8%, 15.7%)	4.2% (1.2%, 10.4%)
	Different grades from 3 graders	0.0%	0.0%	0.0%
**PPOI**	Same grade from 3 graders	90.9% (75.7%, 98.1%)	96.8% (88.8%, 99.6%)	94.7% (88.1%, 98.3%)
	Same grade from 2 graders	9.1% (1.9%, 24.3%)	3.2% (0.4%, 11.2%)	5.3% (1.7%, 11.9%)
	Different grades from 3 graders	0.0%	0.0%	0.0%
**Cirrus 6mm×6mm Macula Scan**				
**MAs**	Same grade from 3 graders	97.1% (84.7%, 99.9%)	90.5% (80.4%, 96.4%)	92.8% (85.7%, 97.0%)
	Same grade from 2 graders	2.9% (0.0%, 15.3%)	9.5% (3.6%, 19.6%)	7.2% (3.0%, 14.3%)
	Different grades from 3 graders	0.0%	0.0%	0.0%
**RI/CD**	Same grade from 3 graders	91.2% (76.3%, 98.1%)	85.7% (74.6%, 93.3%)	87.6% (79.4%, 93.4%)
	Same grade from 2 graders	8.8% (1.9%, 23.7%)	14.3% (6.8%, 25.4%)	12.4% (6.6%, 20.6%)
	Different grades from 3 graders	0.0%	0.0%	0.0%
**CNV**	Same grade from 3 graders	100% (89.7%, 100%)	93.7% (84.5%, 98.2%)	95.9% (89.8%, 98.9%)
	Same grade from 2 graders	0.0%	6.3% (1.8%, 15.5%)	4 (4.1%)
	Different grades from 3 graders	0.0%	0.0%	0.0%
**PPOI**	Same grade from 3 graders	91.2% (76.3%, 98.1%)	90.5% (80.4%, 96.4%)	90.7% (83.1%, 95.7%)
	Same grade from 2 graders	8.8% (1.9%, 23.7%)	9.5% (3.6%, 19.6%)	9.3% (4.3%, 16.9%)
	Different grades from 3 graders	0.0%	0.0%	0.0%
**Cirrus 4.5mm×4.5mm Disc Scan**				
**MAs**	Same grade from 3 graders	94.6% (81.8%, 99.3%)	83.1% (71.7%, 91.2%)	87.3% (79.2%, 93.0%)
	Same grade from 2 graders	5.4% (0.7%, 18.2%)	11 (16.9%)	12.7% (7.0%, 20.8%)
	Different grades from 3 graders	0.0%	0.0%	0.0%
**RI/CD**	Same grade from 3 graders	89.2% (74.6%, 97.0%)	86.2% (75.3%, 93.5%)	87.3% (79.2%, 93.0%)
	Same grade from 2 graders	10.8% (3.0%, 25.4%)	13.8% (6.5%, 24.7%)	12.7% (7.0%, 20.8%)
	Different grades from 3 graders	0.0%	0.0%	0.0%
**CNV**	Same grade from 3 graders	91.9% (78.1%, 98.3%)	87.7% (77.2%, 94.5%)	89.2% (81.5%, 94.5%)
	Same grade from 2 graders	8.1% (1.7%, 21.9%)	12.3% (5.5%, 22.8%)	10.8% (5.5%, 18.5%)
	Different grades from 3 graders	0.0%	0.0%	0.0%
**PPOI**	Same grade from 3 graders	86.5% (71.2%, 95.5%)	90.8% (81.0%, 96.5%)	89.2% (81.5%, 94.5%)
	Same grade from 2 graders	13.5% (4.5%, 28.8%)	9.2% (3.5%, 19.0%)	10.8% (5.5%, 18.5%)
	Different grades from 3 graders	0.0%	0.0%	0.0%
**Maestro2 3mm×3mm Macula Scan**				
**MAs**	Same grade from 3 graders	100% (90.3%, 100%)	93.2% (84.7%, 97.7%)	95.4% (89.6%, 98.5%)
	Same grade from 2 graders	0.0%	6.8% (2.3%, 15.3%)	4.6% (1.5%, 10.4%)
	Different grades from 3 graders	0.0%	0.0%	0.0%

**RI/CD**	Same grade from 3 graders	94.4% (81.3%, 99.3%)	87.7% (77.9%, 94.2%)	89.9% (82.7%, 94.9%)
	Same grade from 2 graders	5.6% (0.7%, 18.7%)	12.3% (5.8%, 22.1%)	10.1% (5.2%, 17.3%)
	Different grades from 3 graders	0.0%	0.0%	0.0%
**CNV**	Same grade from 3 graders	97.2% (85.5%, 99.9%)	93.2% (84.7%, 97.7%)	94.5% (88.4%, 98.0%)
	Same grade from 2 graders	2.8% (0.1%, 14.5%)	6.8% (2.3%, 15.3%)	5.5% (2.1%, 11.6%)
	Different grades from 3 graders	0.0%	0.0%	0.0%
**PPOI**	Same grade from 3 graders	91.7% (77.5%, 98.2%)	94.5% (86.6%, 98.5%)	93.6% (87.2%, 97.4%)
	Same grade from 2 graders	8.3% (1.8%, 22.5%)	5.5% (1.5%, 13.4%)	6.4% (2.6%, 12.8%)
	Different grades from 3 graders	0.0%	0.0%	0.0%
**Maestro2 6mm×6mm Macula Scan**				
**MAs**	Same grade from 3 graders	97.1% (85.1%, 99.9%)	90.5% (81.5%, 96.1%)	92.7% (86.0%, 96.8%)
	Same grade from 2 graders	2.9% (0.1%, 14.9%)	9.5% (3.9%, 18.5%)	7.3% (3.2%, 14.0%)
	Different grades from 3 graders	0.0%	0.0%	0.0%
**RI/CD**	Same grade from 3 graders	91.4% (76.9%, 98.2%)	86.5% (76.5%, 93.3%)	88.1% (80.5%, 93.5%)
	Same grade from 2 graders	8.6% (1.8%, 23.1%)	13.5% (6.7%, 23.5%)	11.9% (6.5%, 19.5%)
	Different grades from 3 graders	0.0%	0.0%	0.0%
**CNV**	Same grade from 3 graders	100% (90.0%, 100%)	93.2% (84.9%, 97.8%)	95.4% (89.6%, 98.5%)
	Same grade from 2 graders	0.0%	6.8% (2.2%, 15.1%)	4.6% (1.5%, 10.4%)
	Different grades from 3 graders	0.0%	0.0%	0.0%
**PPOI**	Same grade from 3 graders	91.4% (76.9%, 98.2%)	94.6% (86.7%, 98.5%)	93.6% (87.2%, 97.4%)
	Same grade from 2 graders	8.6% (1.8%, 23.1%)	5.4% (1.5%, 13.3%)	6.4% (2.6%, 12.8%)
	Different grades from 3 graders	0.0%	0.0%	0.0%
**Maestro2 4.5mm×4.5mm Disc Scan**				
**MAs**	Same grade from 3 graders	100% (90.0%, 100%)	89.9% (80.2%, 95.8%)	93.3% (86.6%, 97.3%)
	Same grade from 2 graders	0.0%	10.1% (4.2%, 19.8%)	6.7% (2.8%, 13.4%)
	Different grades from 3 graders	0.0%	0.0%	0.0%
**RI/CD**	Same grade from 3 graders	94.3% (80.8%, 99.3%)	84.1% (73.3%, 91.8%)	87.5% (79.6%, 93.2%)
	Same grade from 2 graders	5.7% (0.7%, 19.2%)	15.9% (8.2%, 26.7%)	12.5% (6.8%, 20.4%)
	Different grades from 3 graders	0.0%	0.0%	0.0%
**CNV**	Same grade from 3 graders	94.3% (80.8%, 99.3%)	95.7% (87.8%, 99.1%)	95.2% (89.1%, 98.4%)
	Same grade from 2 graders	5.7% (0.7%, 19.2%)	4.3% (0.9%, 12.2%)	4.8% (1.6%, 10.9%)
	Different grades from 3 graders	0.0%	0.0%	0.0%
**PPOI**	Same grade from 3 graders	88.6% (73.3%, 96.8%)	91.3% (82.0%, 96.7%)	90.4% (83.0%, 95.3%)
	Same grade from 2 graders	11.4% (3.2%, 26.7%)	8.7% (3.3%, 18.0%)	9.6% (4.7%, 17.0%)
	Different grades from 3 graders	0.0%	0.0%	0.0%

Abbreviations: Microaneurysms- MAs, Retinal Ischemia/Capillary Dropout- RI/CD, Choroidal neovascularization- CNV, Primary Pathology of Interest- PPOI.
